# Autonomous navigation and active perception with complex articulated AUVs

**DOI:** 10.3389/frobt.2026.1789488

**Published:** 2026-09-11

**Authors:** Marios Xanthidis, Kostas Alexis, Eleni Kelasidi

**Affiliations:** 1 SINTEF Ocean, Trondheim, Norway; 2 Department of Engineering Cybernetics, NTNU, Trondheim, Norway; 3 Department of Mechanical and Industrial Engineering, NTNU, Trondheim, Norway

**Keywords:** active perception, autonomous navigation, obstacle avoidance, planning under uncertainty, underwater robotics

## Abstract

With the recent progress in underwater robotics, autonomous underwater vehicles (AUVs) are increasing their mission capacity and range of applications operating in proximity to underwater infrastructure and closer to the wave zone for data collection and environmental monitoring. Future applications will necessitate more complex articulated systems that will be tasked to operate with increased safety, efficiency, and capacity. In this article, we present a novel motion planning framework for enabling, for the first time, safe autonomous navigation and active perception in high-dimensional articulated AUVs, employed with multiple arbitrary-configured sensors, in complex dynamic environments that are prone to uncertainties, disturbances, and currents. Inspired by previous work, we expand on a locally optimal and empirically safe pipeline designed for holonomic AUVs and enable it to handle articulated AUVs with potentially non-trivial dynamics that are unknown to the planner. We test the proposed methodologies with challenging simulated cases using an underwater snake robot (USR) with realistic dynamics and a generic underwater vehicle-manipulator system (UVMS) comprising of a holonomic base employed with a manipulator. Our results showcase the effectiveness of the proposed technique in producing efficient and safe real-time solutions, while discussing current limitations, computational bottlenecks, and potential solutions.

## 1 Introduction

Underwater autonomy is a domain of constantly growing interest for both academia and industry, with most missions requiring fundamental functionalities, such as autonomous navigation and active perception, for ecosystem monitoring, marine archaeology, resource utilization, infrastructure maintenance, defense, and public safety (Diamanti et al., 2024; Benoit-Bird et al., 2018; Amundsen et al., 2021; Wang Q. et al., 2023; Topini et al., 2023). Such operations pose significant challenges to hardware, state estimation, and motion planning solutions.

Autonomous underwater mapping and navigation are challenging tasks due to the complex underwater environment. The underwater scene is often described as deep, dark, and dangerous, with low visibility and ocean currents in remote areas that introduce significant challenges to the operations (Sans-Muntadas et al., 2022; Xanthidis et al., 2020, 2021b).

**FIGURE 1:**
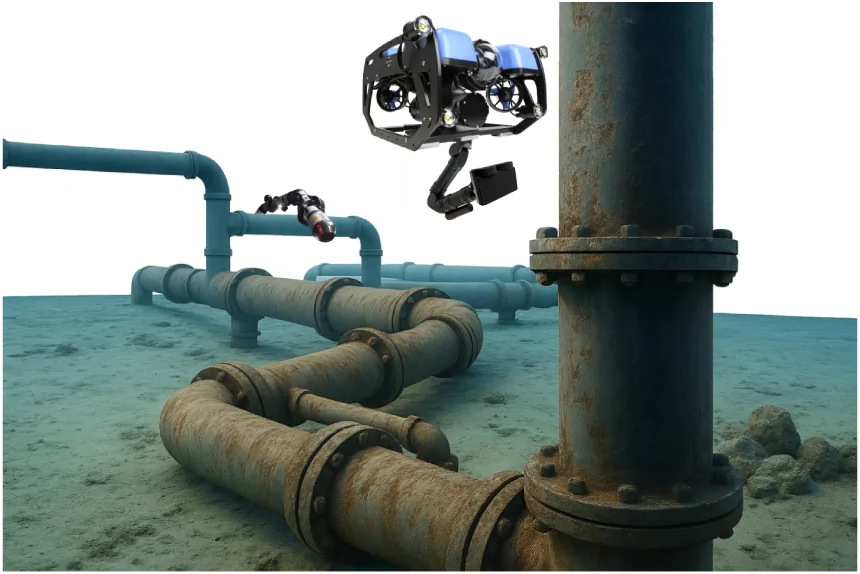
Depiction of multi-articulated underwater robots inspecting benthic infrastructure to prevent ecological damage, which is an aspiration of the proposed work. Two different systems were considered that also inspired the proposed general formulation and validation: As an example, an Eelume M-Series AUV inspecting pipes is shown at the rear of the image, while a BlueROV2 with an Alpha Reach manipulator carrying a sensor, such as a Sonar 3D-15, performs an inspection in close proximity to the infrastructure.

Regarding the motion planning aspect, which is the primary focus of this work, underwater robots are expected to produce motions that respect the kinematic and dynamic abilities of each system and produce safe paths that avoid collisions with static and dynamic structures, and remain resilient in the presence of disturbances in the wave zone, currents, as well as motion and map uncertainty. Meanwhile, robust control under such demanding conditions may require comprehensive analytical models that consider the kinematic and dynamic behavior of the robotic system; something that is often notoriously difficult to obtain for non-trivial platforms. Although methodologies that address the intersection of such issues have been presented recently (Xanthidis et al., 2024), these challenges force most underwater platforms to have simple and well-studied designs with more limited variability in contrast to other domains. Autonomous underwater operations almost exclusively utilize robots of rigid shape (e.g., box- or torpedo-shaped) with sensors mounted in fixed positions, with articulation being present mostly in the context of manipulation for intervention with remotely operated vehicles (ROVs) (Ridao et al., 2015; Khatib et al., 2016). Few works consider articulation for propulsion (Bjørlo et al., 2025; Zhao et al., 2024). Thus, most methodologies for autonomous navigation and active perception in the underwater domain assume rigid non-articulated AUVs, with only a few recent examples investigating a vastly unexplored potential that lies in more complex designs, such as for better data gathering in marine archaeology (Diamanti et al., 2024). Providing motion planning solutions on such complex articulated designs for AUVs not only provides foundational steps for realizing the expected increase in the complexity of underwater systems in the future, capable of multiple tasks and interactions with the environment, but also enables new sensing modalities with variable sensing configurations that allow sensors to move independently from the considered main body of the AUV. This is a particularly important capacity when operating in proximity to dynamic and sensitive ecosystems or infrastructure, as shown in Figure 1.

The motivation for this article is twofold.

First, we address this research gap by providing, to the best of our knowledge, the first autonomous underwater navigation and active perception framework for arbitrary articulated AUVs in challenging underwater conditions. Second, in the process of accomplishing the above, we showcase the importance and the merits of emphasizing generality in autonomy by exploiting system and environment agnosticism and qualitatively scaling up the capacity of such methods. As a base framework, we utilized ResiVis (Xanthidis et al., 2024). ResiVis monitors the performance of the path follower to adaptively decide safe real-time plans mitigating wave zone disturbances, currents, dynamic obstacles, and state, map, and motion uncertainty in rigid holonomic robots, by moving within an analytically computed safety clearance. We leverage the holistic and agnostic nature of ResiVis to include applications for arbitrary articulated AUVs by redefining the optimization problem, reformulating the safe clearance formulation, and providing novel kinematic constraints. The proposed pipeline maintains the holistic and agnostic capacity of ResiVis, extending autonomous navigation and active perception on holonomic or non-holonomic articulated AUVs with unknown dynamics, such as generic holonomic UVMSs or underwater snake robots. This work contributes as follows:

1. A novel holistic, general, agnostic, and adaptive autonomous navigation and active perception framework is presented for arbitrary articulated AUVs moving in arbitrary conditions, potentially perceiving multiple objects of interest.
2. New adaptations that maintain safety and efficiency are introduced, including adaptations for non-holonomic systems with arbitrary complex dynamics, to perform multi-objective active perception.
3. Extensive simulation experiments showcase the effectiveness of the proposed technique in complex static and dynamic environments on two different articulated AUVs: an underwater snake robot (USR) with realistic dynamics, inspired by the Eelume M-Series AUV, and a generic underwater vehicle-manipulator system (UVMS) of a holonomic base employed with a manipulator (Figure 1).
4. Discussion of lessons learned, assumptions, and limitations provides insights into the proposed framework.

## 2 Related work

Despite the multidirectional progress in autonomy over the past years, safe underwater navigation remains a difficult problem of increasing interest, even for rigid AUVs with a common design (Guo et al., 2021; Panda et al., 2020; Kot, 2022). Challenges considered for motion planning involve maneuvering in 3D, robustness against uncertainty and disturbances, and handling of dynamic obstacles. Due to challenges arising from high computational expenses and sensing limitations of 2D acoustic sensors, most navigation pipelines were limited to 2D maneuvering or vertical relief assumptions (Green and Kelly, 2007; Knepper and Mason, 2009; Pivtoraiko et al., 2009; Branicky et al., 2008; Hernández et al., 2016; Vidal et al., 2018; Yuan and Qu, 2009). Fundamental studies have also considered dynamic obstacles in 2D (Fiorini and Shiller, 1998; Fox et al., 1997; Tychonievich et al., 1989), and inspired contributions in the maritime domain (Lin et al., 2019; Benjamin et al., 2019; Zhang et al., 2017).

A substantial amount of research, with the aforementioned limitations, has focused on uncertainty mitigation, especially due to disturbances or currents (Petres et al., 2007; Williams et al., 2001; Moulton et al., 2019; Alvarez et al., 2004; Yilmaz et al., 2008; Caldwell et al., 2010; Subramani and Lermusiaux, 2019; Pairet et al., 2018; Garau et al., 2009).

For 3D navigation, efficient pipelines have been presented for static environments (Tusseyeva et al., 2013; Tan et al., 2004; McMahon and Plaku, 2016; Yu et al., 2019; Wiig et al., 2020; Xanthidis et al., 2020; Pairet et al., 2021; Vidal et al., 2019; Lee et al., 2017; Hernández et al., 2019), dynamic environments (Hao et al., 2022), or for highly-dynamic ones (Amundsen et al., 2024a) with simplified geometries. Notably, ResiPlan (Xanthidis et al., 2023), a framework that also inspired this work, uses fast path optimization to generate trajectories that address the intersection of all these challenges, although it assumes holonomic rigid AUVs and it necessitates the dynamic surroundings to move more slowly than the robot itself. Research that followed resolved the latter limitation using geometric simplifications (Amundsen et al., 2024b) but not the former. Methods for articulated robots moving in 3D have been presented in the aerial domain (Kulkarni et al., 2020) and also underwater in the form of AUVs employed with manipulators (Ribas et al., 2011) or articulated bio-inspired robots (Liljebäck et al., 2014; Kelasidi et al., 2018; Wang et al., 2020; Duraisamy et al., 2019; Shintake et al., 2020; Berlinger et al., 2021; Zhao et al., 2025). Past research has focused on underwater snake robots (USR) and utilized gait adaptation for motion planning, although most are limited to 2D (Hatton et al., 2013; Guo et al., 2018; Hanssen et al., 2020; Hannigan et al., 2020; Li et al., 2021; Liu et al., 2022; Wang L. et al., 2023; Ding et al., 2023; Jiang et al., 2024). Real-time autonomous navigation of such AUVs in 3D has been realized with reactive policies (Hoffmann, 2018), sampling-based techniques (Zhao et al., 2024), and methodologies utilizing motion primitive libraries with path optimization (Bjørlo et al., 2025), although limited attention has been given to handling in parallel the dynamic and uncertain nature of realistic underwater conditions. There is a major challenge for such systems, given their high dimensionality combined with the computationally expensive nature of kinodynamic planning (LaValle, 2011).

Active perception is a problem that was introduced more than four decades ago (Aloimonos et al., 1988; Bajcsy et al., 2018). However, it remains challenging with growing interest in multiple domains and settings. Initial works in 2D focused on state-estimation improvement (Feder et al., 1999; Makarenko et al., 2002; Stachniss et al., 2004; Martinez-Cantin et al., 2007; Rekleitis, 2012; Zhang et al., 2015), inspection (Danner and Kavraki, 2000), or tracking (Gao et al., 2023). Most focus for 3D active perception has been oriented from aerial robotics for tracking a single objective (Potena et al., 2017; Yang et al., 2019; Potena et al., 2019; Sheckells et al., 2016), often in obstacle-free environments (Spica et al., 2017; Costante et al., 2018; Spasojevic et al., 2020; Indelman et al., 2015). Unfortunately, 3D active perception with occlusion-free guarantees in static environments has only been realized offline due to computational expense (Bircher et al., 2016; Zhang and Scaramuzza, 2020; Gemerek et al., 2022), with a notable exception that enabled tracking of a single objective (Penin et al., 2018). The challenge of the problem in 3D has pushed researchers to develop local methods based on low-level control across a planned path in static environments (Falanga et al., 2018; Lee et al., 2020) or in environments with known geometries (Xing et al., 2023). Active perception for articulated robots in dry conditions has been investigated with a strong focus on manipulation (Jauhri et al., 2024).

Active perception for rigid AUVs has been employed for inspection tasks, such as for ship hulls (Hover et al., 2012; Hollinger et al., 2013), and for reducing state uncertainty (Frolov et al., 2014; Chaves et al., 2016), although mostly to an offline extent, or with high computational commands, for real time (Vidal et al., 2020). Real-time pipelines were introduced with a curiosity-based bias for underwater environments with significant free space (Girdhar et al., 2014), for industrial application in aquaculture (Amundsen et al., 2021), for reducing loss of track for vision-based state estimation (Willners et al., 2023), and exploration (Singh et al., 2025). More recently, learning-based techniques have also been tested with successful deployments, although with common limitations for such techniques, such as out-of-distribution inference (Karapetyan et al., 2020; Manderson et al., 2020; Lin et al., 2023; Ruscio et al., 2023). A pipeline that focuses on underwater active perception with drift errors, although without obstacle avoidance, has been presented (Chang et al., 2022). Finally, ResiVis (Xanthidis et al., 2024), our recent previous work, robustified active perception for rigid holonomic systems while guaranteeing real-time safety in challenging cluttered environments with static and dynamic obstacles, wave zone disturbances and errors, currents, and state and motion uncertainty.

To the best of our knowledge, the intersection of the problems addressed by ResiVis, including articulation of underwater platforms with complex and potentially unmodeled kinodynamics, remains an open problem that has yet to be addressed. This article and the following sections provide an approach to address this exact problem by significantly augmenting previous contributions, exploiting generality, and developing a new holistic pipeline that further expands by introducing platform agnosticism with respect to articulation and kinodynamics.

## 3 Problem statement

The main goal of this study is to enable complex multi-articulated underwater robotic systems to navigate safely to a goal, potentially while maximizing observations of certain points of interest. The primary focus of this article is motion planning, while perception, object tracking, and guidance lie beyond our scope. The underwater robotic systems of our focus can move in 3D, control an arbitrary configuration and number of joints, and are equipped with an arbitrary number, configuration, and characteristics of sensors that can observe some target objects of interest, represented with 3D points.

More formally, let’s assume a robot with configuration space $C=X\times Y\times Z\times \Psi_{x}\times \Psi_{y}\times \Psi_{z}\times J_{1}\times \cdots \times J_{m}$ , where X,Y,Z⊆R describe the space of the corresponding position coordinates of the robot, $\Psi_{x},\Psi_{y},\Psi_{z}\subseteq \left(-\pi ,\pi \right]$ describes the space for the roll, pitch, and yaw orientations respectively, and $J_{1},\ldots ,J_{m}\subseteq R$ describes the space corresponding to the m controllable joints, with m≥0 . For simplicity, we assume that all joints are revolute, and they are assembled in an open kinematic chain, although other types of joints (such as prismatic) and other robot configurations do not introduce any additional limitations to the reach of this work.

In addition, let $C=\left\{c_{1},\ldots ,c_{l}\right\}$ describe a set of l≥1 sensors, let $T_{s}=\left\{T_{s}^{c_{1}},\ldots ,T_{s}^{c_{l}}\right\}$ be a set of the corresponding transformations for each c∈C sensor providing its global pose in the state s∈C , $F_{s}={⋃}_{c\in C}F_{s}^{c}$ the union of the field of views of all sensors in state s , and let $V^{t}=\left\{v_{1},\ldots ,v_{k}\right\}$ be a set of k points of interest in $R^{3}$ to be observed at time t , with k≥0 . Finally, $O=\left\{o_{1},\ldots ,o_{r}\right\}$ is the set of static or dynamic obstacles where $V^{t}\subseteq O$ , $s_{1}\in C$ define the initial state of the robot, G , the goal region, is defined as a small closed set around the final state $s_{n}\in C$ , $S=\left\{s_{1},\ldots ,s_{n}\right\}$ is the planned safe path to be followed by the robot reached at projected times $T=\left[t_{1},t_{2},\ldots ,t_{n}\right]$ respectively, $\tilde{S}=\left\{{\tilde{s}}_{1},\ldots ,{\tilde{s}}_{n}\right\}$ is the actual executed path respectively at times $\tilde{T}=\left[{\tilde{t}}_{1},\ldots ,{\tilde{t}}_{n}\right]$ with arbitrary resolution for different values of n>2 , $B_{q}^{t}$ is the 3D volume occupied by object q at time t∈T , and $A_{s}$ is the 3D volume occupied by the robot at state s∈S . The objective of this work can be summarized as follows:

$$f\left(S\right)=\underset{S}{\min}\overset{n-1}{\underset{i=1}{\sum }}\left\|s_{i+1}-s_{i}\right\|+M\left(S\right),$$ (1)

$$s.t.\;\overset{\begin{array}{c} t_{i}\in T\\ {\tilde{s}}_{t_{i}}\in \tilde{S} \end{array}}{⋃}\left(A_{{\tilde{s}}_{t_{i}}}\cap \overset{o_{j}\in O}{⋃}B_{o_{j}}^{t_{i}}\right)=\emptyset ,$$ (2)

with

$$M\left(S\right)=\frac{\left|\left\{s_{i}|\forall s_{i}\in S\wedge F_{s_{i}}\cap V^{t_{i}}=\emptyset \right\}\right|}{n},$$ (3)

where Equation 3 computes the percentage of states not perceiving at least one point of interest, Equation 2 guarantees no collisions and contact with the surrounding obstacles, and Equation 1 minimizes the displacement along the path and the number of states not observing an object of interest. In practice, the goal is to find a plan with minimal path length toward a specified destination that, when followed imperfectly by the robot, will guarantee safety in dynamic environments and maximize the observation of at least a subset of given points of interest. For pure autonomous navigation queries, Equation 3 could be ignored from Equation 1, without loss of formulation consistency.

## 4 Proposed approach

To achieve our objective, we utilize past contributions on safe adaptive underwater navigation and active underwater perception. The core methodology utilized is ResiVis (Xanthidis et al., 2024) configured for articulated AUVs, followed by a novel adaptation for the analytical calculation of the safety clearance, and a new extension accommodating multiple sensing units with arbitrary sensing objectives. This section will continue by providing some basic background information before continuing with the new contributions.

### 4.1 Background on ResiVis

ResiVis is a robust real-time framework for enabling autonomous active perception on holonomic AUVs moving in 3D, while operating in underwater environments with challenging geometry and conditions, such as wave zone disturbances, currents, dynamic obstacles, and state, map, and motion uncertainty. The core optimization process of ResiVis is Trajopt (Schulman et al., 2014), similar to previous work (Xanthidis et al., 2020; Xanthidis et al., 2021b; Xanthidis et al., 2023). Trajopt’s objective function, shown in Equation 4 for a path $S=\left\{s_{1},s_{2},\ldots ,s_{n}\right\}$ , minimizes the path length:

$$f\left(S\right)=\underset{S}{\min}\overset{n-1}{\underset{i=1}{\sum }}\left\|s_{i+1}-s_{i}\right\|.$$ (4)

These constraints are introduced as ℓ1 penalties:

$$H\left(s_{i},s_{i+1}\right)=\underset{o\in O}{\sum }\left|d_{i}^{\mathrm{safe}}-\text{sd}\;\left(\text{cd}\;\left(A_{s_{i}},A_{s_{i+1}}\right),B_{o}^{t_{i+1}}\right)\right|,$$ (5)

where sd (.) is the signed distance function between two 3D convex polytopes, $d_{i}^{\mathrm{safe}}$ is a corresponding clearance, and cd (a,b) indicates the convex hull operation between volumes $a,b\subseteq R^{3}$ , which approximates the swept-out volume of the robot transition between two states. For non-overlapping polytopes, the signed distance function is computed using the Gilbert–Johnson–Keerthi algorithm (Gilbert et al., 1987), while the Expanding Polytope Algorithm (Van Den Bergen, 2001) is used for overlapping polytopes. This formulation is a key attribute contributing to the effectiveness of the methodology, both reducing computational expenses and improving safety. The utilization of swept-out volumes for collision checking, instead of specific states, reduces the number of states needed to represent a path with resolution-dependent safety while, at the same time, it provides continuous time safety guarantees. The latter key feature is utilized to provide solutions with safety guarantees by analytically computing clearances adaptive to the performance of the AUV and the conditions of the environment as introduced in previous work (Xanthidis et al., 2023).

In summary, the discrepancy between planning and acting is monitored by computing the difference between the kinematics assumed during planning and the performance of the path follower:

$$e_{\tau }=\left\|\left\lceil s_{\tau }\right\rceil -\left\lceil {\tilde{s}}_{\tau }\right\rceil \right\|,$$ (6)

where ⌈s⌉ indicates the translational component of the state s , $s_{\tau }$ is the expected position at time τ , and ${\tilde{s}}_{\tau }$ is the corresponding actual position measured. The error is used to calculate a safe clearance that adapts better to the errors experienced. In a sliding window fashion from a time $t_{0}$ to τ , to facilitate worst-case reasoning with temporal locality, given a set of the latest recorded errors $E_{\tau }^{t_{0}}$ , the clearances are computed adaptively for each state $s_{i}$ :

$$d_{i}^{\mathrm{safe}}=\alpha \;\max\left(E_{\tau }^{t_{0}}\right)+\epsilon ,$$ (7a)

$$\;+\frac{\alpha \;\max E_{\tau }^{t_{0}}\beta av\left(S\right)}{\left\|u_{r}\right\|\Delta t^{\max\left(E_{\tau }^{t_{o}}\right)}},$$ (7b)

$$\;+\gamma \underset{u_{o}\in U_{O}^{i}}{\max}\left(\left\|u_{o}\right\|\right)\underset{t\in \Delta T_{\tau }^{t_{0}}}{\max}\left(t\right)\frac{\beta av\left(S\right)}{\left\|u_{r}\right\|\Delta t^{\max\left(E_{\tau }^{t_{o}}\right)}},$$ (7c)

$$\;+P\left(t_{i}\right).$$ (7d)

Here, ϵ>0 is the minimum possible clearance, α,β,γ≥0 are parameters representing the percentage of potential increased future errors, path length, and the obstacles’ velocities, respectively, $\Delta t^{\max\left(E_{\tau }^{t_{o}}\right)}$ is the maximum replanning duration in the given timeframe, av(S) is the average distance between two consecutive states along the path, $u_{r}$ is the expected velocity of the robot, $U_{O}^{i}$ is a set of measured velocities of the obstacles in the surroundings, and P(.) is an estimate of the growing uncertainty at time $t_{i}$ in the case of a dead-reckoning estimation.

The Equation 7a term ensures safety for zero-mean errors, such as typical control errors and oscillations due to waves, by maintaining at least a distance equal to the maximum error experienced from previously recorded errors. To continue, adding Equation 7b enables safety for persistent errors, such as currents or other persistent forces of arbitrary direction, that affect the motion of the robot. The intuition behind this term is to assume that all errors experienced in the past were solely a result of worst-case persistent forces perpendicular to the desired motion of the robot. Thus, the clearance must increase further to move the robot toward a direction that its velocity vector would counter the maximum velocity of the assumed persistent forces. This assumption results in more conservative performance when most of the errors are not due to currents or other persistent forces. If the velocity of the current $u_{f}$ is known, replacing $\frac{\alpha \;\max E_{\tau }^{t_{0}}}{\Delta t^{\max\left(E_{\tau }^{t_{o}}\right)}}$ with $u_{f}$ will provide a tighter bound. Equation 7a and Equation 7b ensure safety with static surroundings.

Equation 7c increases the clearance to accommodate nearby moving obstacles, holding hard safety guarantees for obstacles moving slower than the combined velocity of the robot and the current. In practice, it is a combination of Equations 7a, b applied from the perspective of the robot, further increasing the distance from the obstacles projecting their expected future potential positions. The final term, Equation 7d, provides safety against the expected uncertainty increase, in case of employing dead-reckoning due to localization failures (Joshi et al., 2023).

Proofs of the strong safety guarantees and more details could be found in the original ResiPlan article (Xanthidis et al., 2023). To enable active perception, ResiVis utilizes geometric constraints by projecting line segments in front of the sensors matching the expected sensing range and minimizing the distance to the nearby object of interest o∈V . For a sensor c∈C , if $d_{min}^{c}$ and $d_{max}^{c}$ are the minimum and maximum desired sensing distance, $T_{w}^{s}$ is the transformation of the robot in the world frame, and $T_{r}^{c}$ is the transformation of the sensor c in the local robot frame. If $p_{min}^{c}={\left[d_{min}^{c}\text{ 0 0 }1\;\right]}^{T}$ and $p_{max}^{c}={\left[d_{max}^{c}\text{ 0 0 }1\;\right]}^{T}$ , the sensor representation is defined as follows:

$$F_{s}^{∼}=\underset{c\in C}{⋃}\left\{\bar{\left\lceil T_{w}^{s}T_{r}^{c}p_{min}^{c}\right\rceil \left\lceil T_{w}^{s}T_{r}^{c}p_{max}^{c}\right\rceil }\right\},$$ (8)

where $\bar{p_{1}\;p_{2}}$ represents the line segment between the two 3D points $p_{1}$ and $p_{2}$ . The geometric constraint then for every state s and a weight w is given by

$$Vis\left(s\right)=w\underset{\begin{array}{c} o\in V\\ \bar{f}\in F_{s}^{∼} \end{array}}{\min}dist\left(\bar{f},\left\lceil o\right\rceil \right).$$ (9)

The above formulation is adapted for moving objects of interest by projecting their future position. Note that the current formulation does not effectively deal with occlusions. More details are included in the original ResiVis article (Xanthidis et al., 2024).

### 4.2 Proposed adaptations for articulated AUVs

#### 4.2.1 Optimization process formulation

Trajopt (Schulman et al., 2014), the core planner, as described above, has been utilized successfully to produce real-time high-quality paths in the underwater domain (Xanthidis et al., 2020; Xanthidis et al., 2021b; Xanthidis et al., 2023; Xanthidis et al., 2024). Interestingly, to the best of our knowledge, this is the first time in the underwater domain that Trajopt will be used with systems for which it was developed, such as high-dimensional articulated robots. In the previous contributions, the collision constraint, described in Equation 5, holds safety guarantees for a rigid-body robot because the validity of the approximation of the swept-out volumes in the in-between transitions depends on the robots maintaining their shape.

Rigid approximations for collision checking could provide a significant boost in highly articulated robots. A safe rigid approximation should be a volume in which any joint configuration would be included. This can potentially result in extremely conservative volumes that limit the reachable workspace of such systems. Thus, for the sake of completeness and operability, in this article, the full articulated model is utilized. If $A_{s}^{l_{j}}$ represents the 3D volume of the link controlled by the j joint (0<j≤m) and $A_{s}^{l_{0}}$ corresponds to the assumed base link $l_{0}$ :

$$A_{s}=\overset{m}{\underset{l=0}{⋃}}A_{s}^{l}.$$ (10)

Then, by substituting Equation 10 into Equation 5, the more general form of the collision constraint with the decomposed volume of the robot takes the following form:

$$\begin{array}{cc} H\left(s_{i},s_{i+1}\right) & =\underset{o\in O}{\sum }\overset{m}{\underset{j=0}{\sum }}\left|d_{i}^{\mathrm{safe}}-\text{sd}\;\left(\text{cd}\;\left(A_{s_{i}}^{l_{j}},A_{s_{i+1}}^{l_{j}}\right),B_{o}^{t_{i+1}}\right)\right|\\ & \;+\overset{m-1}{\underset{j=0}{\sum }}\overset{m}{\underset{j^{\prime }=j+1}{\sum }}\left|d_{i}^{\mathrm{safe}}-\text{sd}\;\left(\text{cd}\;\left(A_{s_{i}}^{l_{j}},A_{s_{i+1}}^{l_{j}}\right),\text{cd}\;\left(A_{s_{i}}^{l_{j^{\prime }}},A_{s_{i+1}}^{l_{j^{\prime }}}\right)\right)\right| \end{array}.$$ (11)

The first term of Equation 11 ensures collision avoidance between the swept-out volume of each link of the articulated robot and the obstacles. The second term addresses self-collisions by considering the swept-out volumes for each pair of links between two consecutive states. The new formulation is configured and processed by Trajopt’s optimization, utilizing inverse kinematic solvers.

In accordance with our underlying motivation in this work, this is a clear example of how choosing general methodologies with the capacity to scale up well provides long-term advantages. Even if other methodologies could intuitively appear to be more appropriate, given that powerful general techniques may be underutilized in the initial target application, they could unlock and facilitate easier transitions to solutions directed to more complex applications, in contrast to methods that may lack the capacity for generalization.

#### 4.2.2 Active perception constraints

In ResiVis, active perception is achieved by formulating a computationally efficient approximation of the union of the field of views of each sensor, shown in Equation 8, and then by applying the geometric constraints in Equation 9. Due to embracing generality, the formulation presented is already capable of accommodating multiple arbitrarily placed heterogeneous sensors in rigid-body AUVs, which has already been investigated (Xanthidis et al., 2021a). However, the sensors are assumed to be fixed on the AUV’s main frame, while in articulated AUVs, the sensors are expected to move with respect to the robot’s frame. The key term that requires attention for adapting the given formulation to articulated systems is $T_{r}^{c}$ in Equation 9, describing the relative transformation between the main robot frame r and the sensor c . Similarly, this transformation for articulated systems could be found by solving the forward kinematics for the end effectors corresponding to each sensor. Let $L_{a}^{b}=\left\{l_{k_{0}},l_{k_{1}},l_{k_{2}},\ldots ,l_{k_{h}}\right\}$ define the kinematic chain from link $l_{a}$ to link $l_{b}$ , as a set of consecutive links, with $k_{0}<k_{1}<\ldots <k_{h}\leq m$ , 0≤h≤m , $k_{0}=a$ , and $k_{h}=b$ . Then, the kinematic chain from the base link $l_{0}$ to the a sensor link $l_{c}$ for any sensor c is given by $L_{0}^{c}$ with $k_{0}=0$ and $l_{k_{h}}=l_{c}$ . If $T_{a}^{b}$ describes the transformation between two consecutive links, from link a to link b , $T_{r}^{c}$ is computed as follows:

$$T_{r}^{c}=\overset{m-1}{\underset{i=0}{\prod }}T_{l_{k_{0}}}^{l_{k_{i+1}}}=T_{l_{0}}^{l_{k_{1}}}\cdot T_{l_{k_{1}}}^{l_{k_{2}}}\cdot \cdots \cdot T_{l_{k_{h-1}}}^{l_{c}}.$$ (12)

Then, application of Equation 11 in Equation 8, and subsequently in Equation 9, maintains consistency during optimization and extends active perception to arbitrary articulated systems.

The additional degrees of freedom between the different sensors also allow for more efficient solutions, utilizing the potential heterogeneity of each sensor, but also for multi-objective active perception by assigning different objectives to different sensors. Such an example could be utilizing one sensor to always observe the direction of motion of the robot, in order to register obstacles and inform obstacle avoidance, while the other sensor is assigned to perceive objectives in arbitrary directions. This is an important new potential that addresses sensing limitations in previous works (Xanthidis et al., 2024). We investigate this potential in the experiments of the next section.

A common approach that is chosen in mobile manipulator systems to improve computation of motion planning solutions is to reduce the dimensionality by treating the main mobile base of the robot and the manipulators independently. We decided to treat both these components as a unified system because (1) we would like to challenge the proposed approach providing a solution that is generic and avoids system-specific optimizations, and, more importantly, (2) because in contrast to in-air or in-space conditions, the underwater environment suffers from turbidity, limiting the sensing range of the optical sensor; thus, the base and the manipulator should be co-optimized to maintain desired sensing distances that may be shorter.

Please note that occlusions due to the presence of other objects are beyond the scope of this work and are left as a topic of future research.

The current formulation assumes that for the sensors considered, sensing benefits by centering the target objects in the sensors’ field of view, something that may not be universally applicable for sensors with characteristics other than cameras or directional sonars, or for applications that can benefit from peripheral data collection. Although the proposed formulation may be utilized to address such sensing needs by increasing the number of the projected line segments, we leave the potential for a more comprehensive approximation of the field of view as future work.

#### 4.2.3 Adaptive clearance improvement

As introduced at the beginning of this section, a key feature of ResiVis is its robust adaptive capacity and the strong safety guarantees obtained by holistically extrapolating the recorded difference between the planned and the measured trajectory during execution. The error is trivially calculated by Equation 6 and the adaptive safety clearance computed by Equations 7a–d, for rigid AUVs.

Please note that, as shown in previous work (Xanthidis et al., 2023), the strong safety guarantees hold with the assumption that the experienced errors up to this point are representative of those to be experienced in the future with bounded expected outliers. On the other hand, in articulated AUVs, the safety guarantees collapse due to the transformation of additional moving links, not represented sufficiently with the main robot frame, and the additional uncertainty of the joints. To respect the assumptions of the safety guarantees of Equations 7a–d, the first two terms, Equations 7a, b, must be modified to accommodate joint errors, while the latter terms, Equations 7c, d, depend only on either external factors of the surroundings or growing uncertainty and need no further care.

To start, Equation 7a was responsible for capturing zero-mean errors related to bounded localization and motion errors. A new error metric is required that also includes the joint displacement error between the planned configuration and the measured state, while it respects the safety assumptions described by Xanthidis et al. (2023), necessitating a rigid holonomic robot. The key idea for the new error calculation is to utilize worst-case reasoning and adapt the clearance with respect to the capacity of the robot to change shape, due to articulation, by finding the maximum displacement of the robot’s volume.

If $A_{s}$ is the volume of the robot at state s of the path, $\tilde{s}$ is the corresponding measured state of the robot when it should be at s (assuming no errors), and two 3D points $p\in A_{s}^{l_{j}}$ and $p^{\prime }\in A_{\tilde{s}}^{l_{j}}$ for any 0≤j≤m so that in the local frame of the j link, $p\equiv p^{\prime }$ holds, then the new proposed error $\overset{link}{e_{\tau }}$ is:

$$\overset{link}{e_{\tau }}=\underset{\begin{array}{c} p\in A_{s}^{l_{j}}\\ p^{\prime }\in A_{\tilde{s}}^{l_{j}} \end{array}}{\max}\left\|p^{\prime }-p\right\|.$$ (13)

The above formulation of Equation 13 attempts to extract the maximum displacement a volume of any link can have by finding the maximum distance between two points that represent the same rigid space in the local frame of the link to which they belong. The computation could be significantly improved by representing each link with corresponding points, generalizing further the previous formulation. Thus, borrowing the formulation from Section 4.2.1, if $P^{l}=\left\{p_{1},p_{2},\ldots \;\right\}$ is a set of representative 3D points for a link l in its local reference frame, $P_{s}^{l}$ is the corresponding points at state s in the world frame, after they have been transformed by $T_{w}^{s}T_{s}^{l}\left[p_{1}\;p_{2}\;\ldots \right]$ , and $P_{s}={⋃}_{k=0}^{m}P_{s}^{l_{k}}$ is the set of all representative points at state s . The error computation then is given by:

$$\overset{link}{e_{\tau }}=\underset{\begin{array}{c} p_{i}\in P^{s}\\ p_{i}^{\prime }\in P^{\tilde{s}} \end{array}}{\max}\left\|p_{i}^{\prime }-p_{i}\right\|.$$ (14)

To further improve computation without loss of accuracy, we represent the base link with its centroid, as in previous works (Kelasidi et al., 2014). For each different link, we define representative points at the center of each revolute joint and at the edge of each end effector, given that no moving parts exist in between and the maximum displacement of a link supported by a revolute joint is expected to be found at the point with the largest distance from the rotational motion. The new error computed in Equation 14 is then applied to Equation 7a and more accurately informs the clearance calculation.

To continue, Equation 7b utilizes the measured error to increase the safety clearance with worst-case reasoning by assuming that the entirety of the error is the result of persistent forces. Such forces in articulated AUVs might be currents or other unmodeled external forces, but they might also be the result of unmodeled dynamics; with the latter being the first time this is considered within the context of general holistic and agnostic closed-loop frameworks with adaptive clearance. The error, Equation 14, already considers the displacement of the robot due to bounded errors on the robot’s and joints’ motion and due to currents or unmodeled dynamics and could be directly utilized in Equation 7b.

**FIGURE 2:**
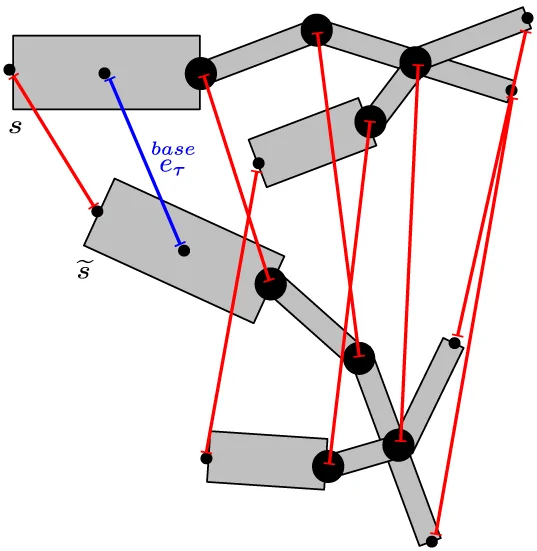
Depiction of the proposed error calculation for the adaptive clearance calculation. An example of a generic articulated system is shown in the desired state s and the actual state achieved $\tilde{s}$ . The blue line shows the distance error assumed for persistent errors $\overset{base}{e_{\tau }}$ , while the red lines show the distances used for computing $\overset{link}{e_{\tau }}$ .

Unfortunately, Equation 7b, due to its conservative nature, is the largest contributor in the clearance computation and sometimes significantly increases the safety clearance. To mitigate potential challenges related to this limitation, given that the joints’ displacement by itself does not necessarily capture exclusively persistent forces, for the calculation of Equation 7b, similar to previous implementations as shown in Equation 6, the following error is adapted to the new notation:

$$\overset{base}{e_{\tau }}=\left\|{\tilde{p}}_{0}-p_{0}\right\|,$$ (15)

where $p_{0}$ and ${\tilde{p}}_{0}$ are the representative points (centroids) of the base link in the expected and measured states, respectively. The idea behind this choice is that unmodeled dynamics with persistent effects on the motion of the AUV will be captured directly simply by the displacement of the base link, which is an intuition that is confirmed in the exhaustive simulation experiments of the next section. Figure 2 depicts an example of the errors introduced for a generic articulated platform.

Combining all the above, for error sets $\overset{link}{E_{\tau }^{t_{0}}}$ and $\overset{base}{E_{\tau }^{t_{0}}}$ corresponding to collection of errors computed by Equations 14, 15 respectively, substituting into Equations 7a–d, the proposed new analytical clearance computation is the following:

$$d_{i}^{\mathrm{safe}}=\alpha \;\max\left(\overset{link}{E_{\tau }^{t_{0}}}\right)+\epsilon ,$$ (16a)

$$\;+\frac{\alpha \;\max\overset{base}{E_{\tau }^{t_{0}}}\beta av\left(S\right)}{\left\|u_{r}\right\|\Delta t^{\max\left(\overset{base}{E_{\tau }^{t_{o}}}\right)}},$$ (16b)

$$\;+\gamma \underset{u_{o}\in U_{O}^{i}}{\max}\left(\left\|u_{o}\right\|\right)\underset{t\in \Delta T_{\tau }^{t_{0}}}{\max}\left(t\right)\frac{\beta av\left(S\right)}{\left\|u_{r}\right\|\Delta t^{\max\left(E_{\tau }^{t_{o}}\right)}},$$ (16c)

$$\;+P\left(t_{i}\right).$$ (16d)

#### 4.2.4 Representative instances of UVMSs

**FIGURE 3:**
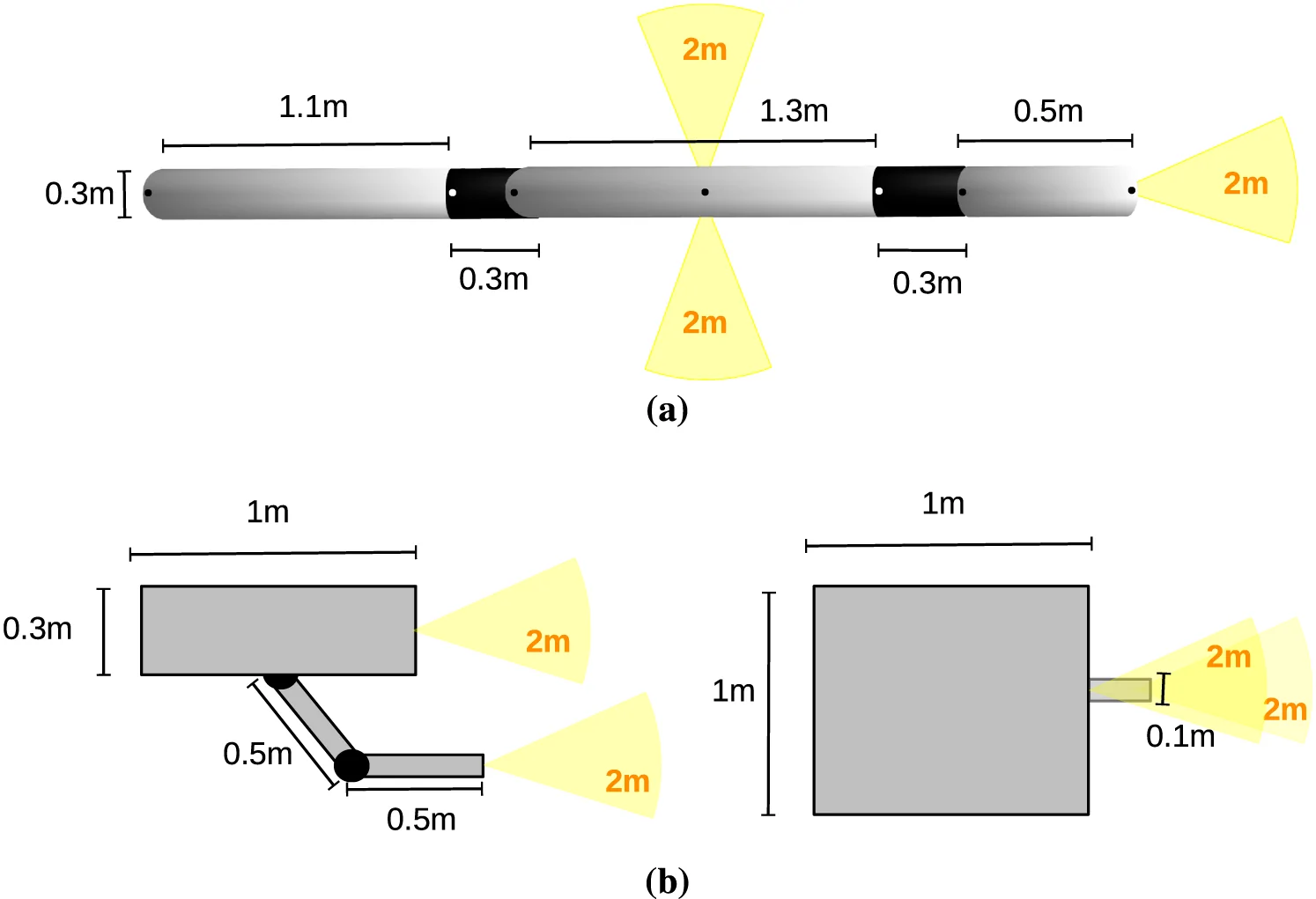
The dimensions and sensor placement of the example articulated AUVs considered while showcasing the proposed formulation and validation, for Snake-M in **(a)** and for ROV-UVMS in **(b)** with side and top–down views.

To continue with the proposed methodology, we focused on two different representative AUV configurations (Figure 3) for clarity of description and experimentation, although it remains highly agnostic system-wise.

- Snake-M: The first platform considered represents non-holonomic UVMSs with complex dynamics, including bio-inspired systems. It is inspired by the Eelume M-series AUV^1^, although with custom dimensions and configuration for the purpose of this article. We utilized a simulated version and chose a total length of 3.5 m consisting of cylindrical components of radius 0.5 m and lengths 0.5 m, 1.3 m, and 1.1 m from front to back, respectively. These modules are connected with spherical joints of length 0.3 m and maximum angular displacement of 30° . Similar to previous work (Kelasidi et al., 2014), uniform density is assumed across each link, so that each link’s center of mass coincides with the center of the link. The center of mass of the middle link is considered the origin frame. For active perception, the sensors are placed on the left and the right of the origin. The simulated model utilized is moving with its articulation and a thruster placed on the front.
- ROV-UVMS: The second platform represents a generic holonomic configuration, similar to a typical ROV, equipped with a two-link manipulator with a length of 0.5 m, connected with spherical joints of 0.2 m. All the segments have a width of 0.05 m. The mobile base has a box shape of 1.0 m × 1.0 m × 0.3 m. The manipulator is mounted in the center of the bottom side of the mobile base. For active perception, two sensors were placed at the end effector and at the center of the front side of the mobile platform. The mobile platform is controllable in the three translational axes and the yaw heading. We decided to model a less redundant manipulator to challenge the proposed planner, given that active coordinated motion of the mobile manipulator is necessary for the desired placement of the end-effector sensor, introducing more potential conflicts.

**FIGURE 4:**
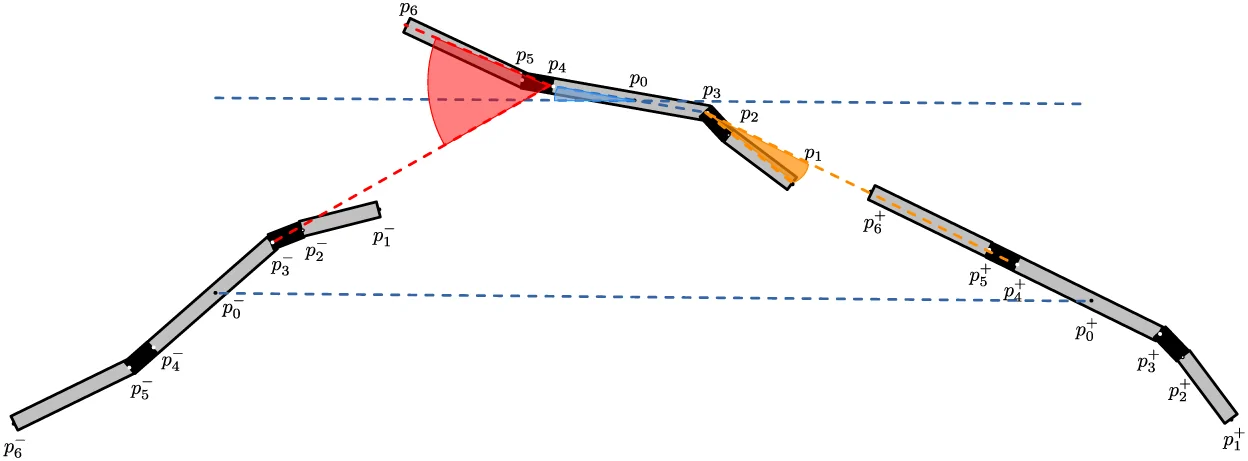
An example of three consecutive states during optimization $s_{i-1}$ , $s_{i}$ , and $s_{i+1}$ from left to right. For state $s_{i}$ , the angles of Equation 18 are shown with blue depicting the first, red depicting the second, and orange depicting the last term.

#### 4.2.5 Kinematic constraints for underwater robotic snakes

With the previous novel enhancements, ResiVis has been generalized to systems with arbitrary articulation and unmodeled kinodynamics with no explicit restrictions. However, as can be deduced from Equations 16a-d, a larger discrepancy between the planned solution and the one executed leads to more conservative behavior by leaving a larger distance from the nearby obstacles. Thus, a motion planning solution that is closer to the expected motion of the robot and better approximates its dynamics will lead to less conservative solutions with a shorter, yet equally safe, clearance.

By default, ResiPlan and ResiVis are completely agnostic on both the kinematics and dynamics of the target system, and they assume holonomic kinematics with no dynamic effects. Any deviations from the default assumptions due to unmodeled kinodynamic effects are treated as “persistent errors” that appear due to system uncertainty affecting the motion, as any other unmodeled persistent source of error would, such as currents. An optional improvement for highly non-holonomic systems with challenging kinodynamics (e.g., underwater snake robots) could be in the form of introducing additional kinematic constraints approximating the expected kinodynamic behavior of these systems. Even rough approximations can significantly improve computation times and reduce conservatism.

There are diminishing effects of adding the complex kinodynamic constraints in the optimization process, due to the increasing probability of local minima issues and increasing decision time due to the significant increase in the computational expense (Hannigan et al., 2020). A negotiation toward balancing the two competing objectives of overly conservative paths and computationally efficient optimization is using additional geometric constraints, adapted within some proximity to the expected motion of the system. As expected, the specific choice of geometric constraints is closely tied to the intricacies of different systems.

In regard to the two systems considered in Figure 3, the ROV-UVMS is assumed to be holonomic; thus, no additional geometric constraints are needed, given that the produced path could be followed perfectly assuming perfect state estimation and control. On the other hand, Snake-M follows curved non-holonomic paths, with the thrusted kinematic model assumed. A solution from the optimization without kinematic constraints may find that the states are considered at arbitrary orientations, and the joints are considered at angles that are not aligned with the curve followed. To produce solutions aligning the links of the robot with the expected curve, simple and easily computed constraints were added.

To start, we set the roll attitude of the robot at states $\psi_{s}\in \Psi$ to zero, as shown in Equation 17:

$$NoRoll\left(s\right)=\psi_{s}=0.$$ (17)

Then, to align the links as shown in Figure 4, let $P_{s_{i}}=\left\{p_{0},p_{1},p_{2},p_{3},p_{4},p_{5},p_{6}\right\}$ , where $p_{0}$ is the centroid, $p_{3}$ and $p_{4}$ are the front and back edges of the base link, $p_{2}$ and $p_{3}$ are the front and back edges of the front connector, $p_{1}$ and $p_{2}$ the front and back edges of the front link, $p_{4}$ and $p_{5}$ the front and back edges of the back connector, and $p_{5}$ and $p_{6}$ the front and back edges of the back link. If $P_{s_{i}}^{-}$ indicates the representative points of the previous state $P_{s_{i-1}}$ , $P_{s_{i}}^{+}$ for the next state $P_{s_{i+1}}$ , and angle(a,b) returns the minimum angle between two line segments a and b translated at the common origin, then the proposed geometric constraint applied is:

$$\begin{array}{ll} & Align\left(s\right)=w_{1}\cdot angle\left(\bar{p_{3}p_{4}},\bar{p_{0}^{-}p_{0}^{+}}\right)+w_{2}\cdot angle\left(\bar{p_{3}p_{1}},\bar{p_{3}p_{4}^{+}}\right)\\ & \;+w_{3}\cdot angle\left(\bar{p_{4}p_{6}},\bar{p_{3}^{-}p_{4}}\right)=0. \end{array}$$ (18)

By minimizing the angles, the first term orients the base link with the line connecting the centroid of the base links for the previous and the next states, aligning it with the curve using the centroids of the previous and next state, which are at equal distance from the state s , matching the intermediate attitude. The last two terms align the front and back links by pointing directly to the next and the previous states. The weights $w_{1}$ , $w_{2}$ , and $w_{3}$ are used to regulate the tolerance of achieving the desired alignment, and, in our experience, required no significant tuning. We applied the above constraints for all states, excluding the initial and goal states, that are already aligned with respect to the dynamics of the AUV.

## 5 Experimental validation

### 5.1 Experimental setup

The proposed pipeline was tested in simulation on its capacity to generate safe paths enabling multi-objective active perception and compared with a non-adaptive baseline maintaining a constant clearance of 0.5 m, similar to previous work (Xanthidis et al., 2020; Xanthidis et al., 2023). The two platforms, shown in Figure 3, were considered during the simulated tests in challenging environments of static and dynamic obstacles, with simulated errors: 1) a simulated underwater snake robot configuration (Snake-M), inspired by the Eelume M-Series, with thrusters, realistic kinodynamics, and 2) a holonomic articulated AUV consisting of a box-like main frame equipped with a manipulator (ROV-UVMS). To further study the capacity of the proposed method to handle complex systems with unmodeled unknown dynamics and provide safe solutions in environments of challenging geometries and conditions, additional experiments are provided for the Snake-M AUV focusing on autonomous navigation.

Embracing the holistic and adaptive nature of the proposed enhancements, our active perception implementation automatically switches to autonomous navigation mode when no sensing objectives are provided by simply not introducing the proposed active perception constraints without any tuning. Both platforms are assumed to know only the current position of the obstacles, while the velocity of the dynamic obstacles is extrapolated by tracking.

Self-collisions are considered in all reported experiments, and in our experience, no observable performance boost was noted by avoiding such collision checking. For the Snake-M experiments, the platform is modeled with a single thruster in the front to decrease maneuverability and increase the dynamic complexity. The robot is controlled using line of sight (LOS) guidance (Breivik and Fossen, 2009) that drives the robot toward a desired waypoint using the heading link with the thruster, while the rest of the kinematic chain follows. Regarding the holonomic articulated AUV (ROV-UVMS), the ideal motion was produced by simple linear interpolation toward the next state. Stochastic errors are added to these kinematic models for our experiments.

All environments were tested with three different error models, simulating variable errors expected to be encountered due to wave zone conditions, localization and motion uncertainty, and currents:

- *Error Free*: Perfect conditions assumed with no errors. Any discrepancy between the planned and executed trajectories was due to unmodeled dynamics.
- *Medium*: Additional random uniform errors in the range of [−0.1,0.1] m in X , Y , and Z , a constant additive error of 0.1 m/s on Y simulating vertical current, and a random uniform error in the range of [−2.5°,2.5°] for roll $\Psi_{x}$ , pitch $\Psi_{y}$ , yaw $\Psi_{z}$ , and each joint angle $J_{1},J_{2},\ldots ,J_{m}$ .
- *High*: Random uniform errors in the range of [−0.2,0.2] m in X , Y , and Z , a constant additive error of 0.2 m/s on Y simulating vertical current, and a random uniform error in the range of [−5°,5°] for roll $\Psi_{x}$ , pitch $\Psi_{y}$ , yaw $\Psi_{z}$ , and each joint angle $J_{1},J_{2},\ldots ,J_{m}$ .

A velocity of 1 m/s was set for both AUVs, while the distance between each state av(S) was set at 3 m for ROV-UVMS and 4 m for Snake-M, due to the increased size of the latter. Regarding parameter tuning, ϵ was set to 0.01 m, while α , β , and γ were set to 1.1 allowing a 10% of increase in errors in future measurements. The same parameters were set for both robots, emphasizing further how the proposed technique can generalize to very different systems.

To challenge the replanning capacity of the developed planner in long path lengths, instead of using receding horizon and reducing the computational cost, the entire path to the goal position was considered. In all experiments, the distance of the goal from the initial position is 50 m. Note that although box-shaped obstacles were used for simplicity to construct the environments, as shown in previous works (Xanthidis et al., 2020), structures of arbitrary geometries can be considered as a collection of convex polytopes. Finally, both the main pipeline, along with the simulator which utilized the OpenRave environment (Diankov and Kuffner, 2008), were run simultaneously on a machine with an Intel ® Core i7-7500 processor (2.70 GHz) and 7.7 GB RAM.

### 5.2 Autonomous navigation simulations with the Snake-M AUV

The goal of the autonomous navigation experiments of this section is to test the capacity of the proposed enhanced planner to safely and efficiently navigate complex cluttered environments, handle multiple dynamic obstacles, mitigate different water conditions, and finally adapt online to expected errors from unknown to the planner’s kinodynamics. For this purpose, three environments were created and tested for different conditions simulated by the three different error models:

- *m1* simulates an environment with highly complex geometry, such as navigation through underwater infrastructure in a disaster scenario. It consists of 13 static obstacles placed in variable orientations, creating narrow free passages of different shapes.
- *m2* consists of seven static vertical obstacles, placed in a way that significant horizontal maneuverability is required.
- *m3* includes 50 randomly distributed obstacles of randomized shape with lengths ranging from 0.1 m to 1 m, random constant velocities from 0.05 m/s to 0.2 m/s, each moving on unknown trajectories formed by six randomly chosen waypoints.

**FIGURE 5:**
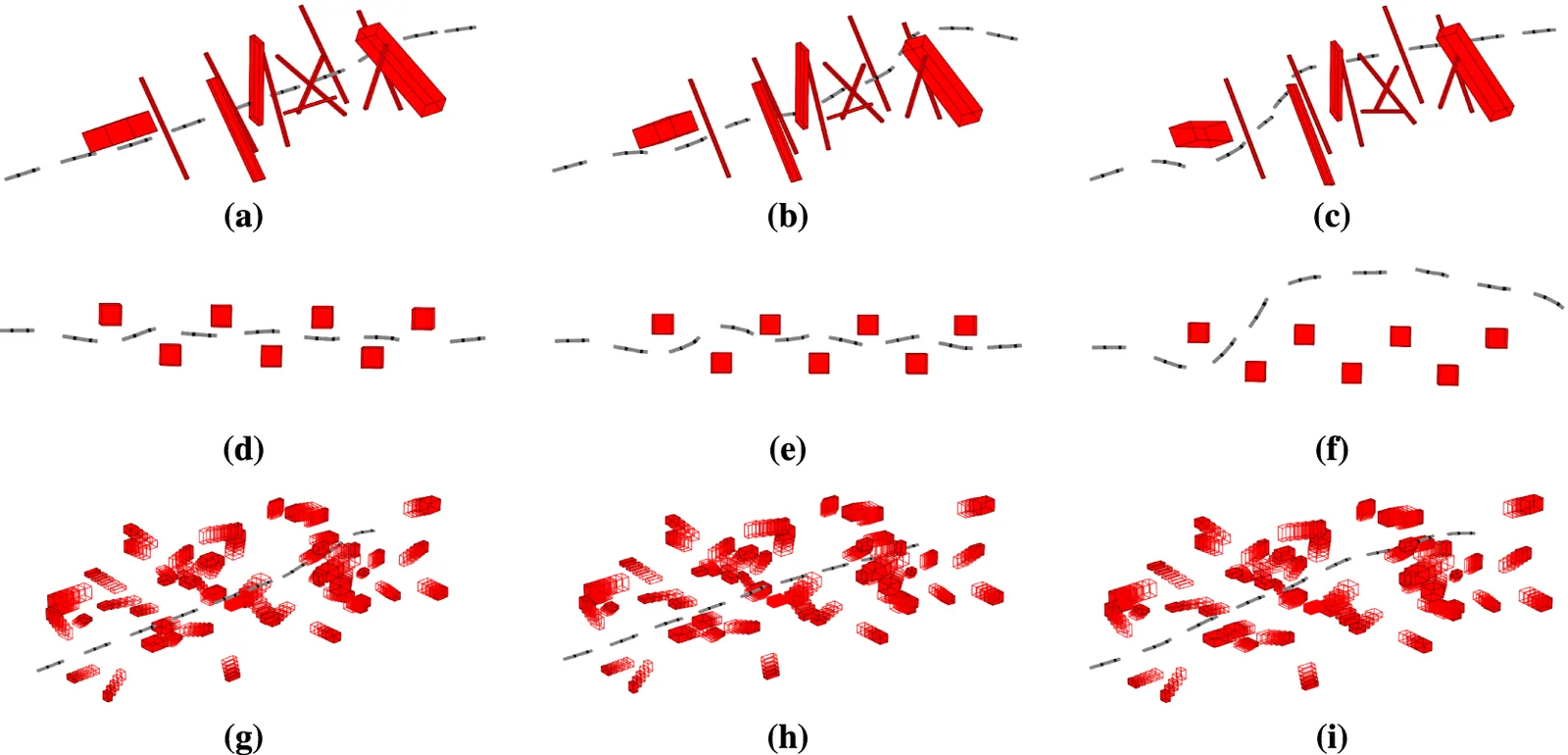
Snapshots of the ResiPlan executions in the test environments (*m1*: **(a–c)**, *m2*: **(d–f)** and *m3*: **(g–i)**) from top to bottom and for different conditions (*Error Free*: **(a,d,g)**, *Medium*: **(b,e,h)**, and *High*: **(c,f,i)**) from left to right, with obstacles shown in red. The velocity and direction of the dynamic obstacles in *m3* are indicated with the fading pattern showing the motion for the first 20 s.

**FIGURE 6:**
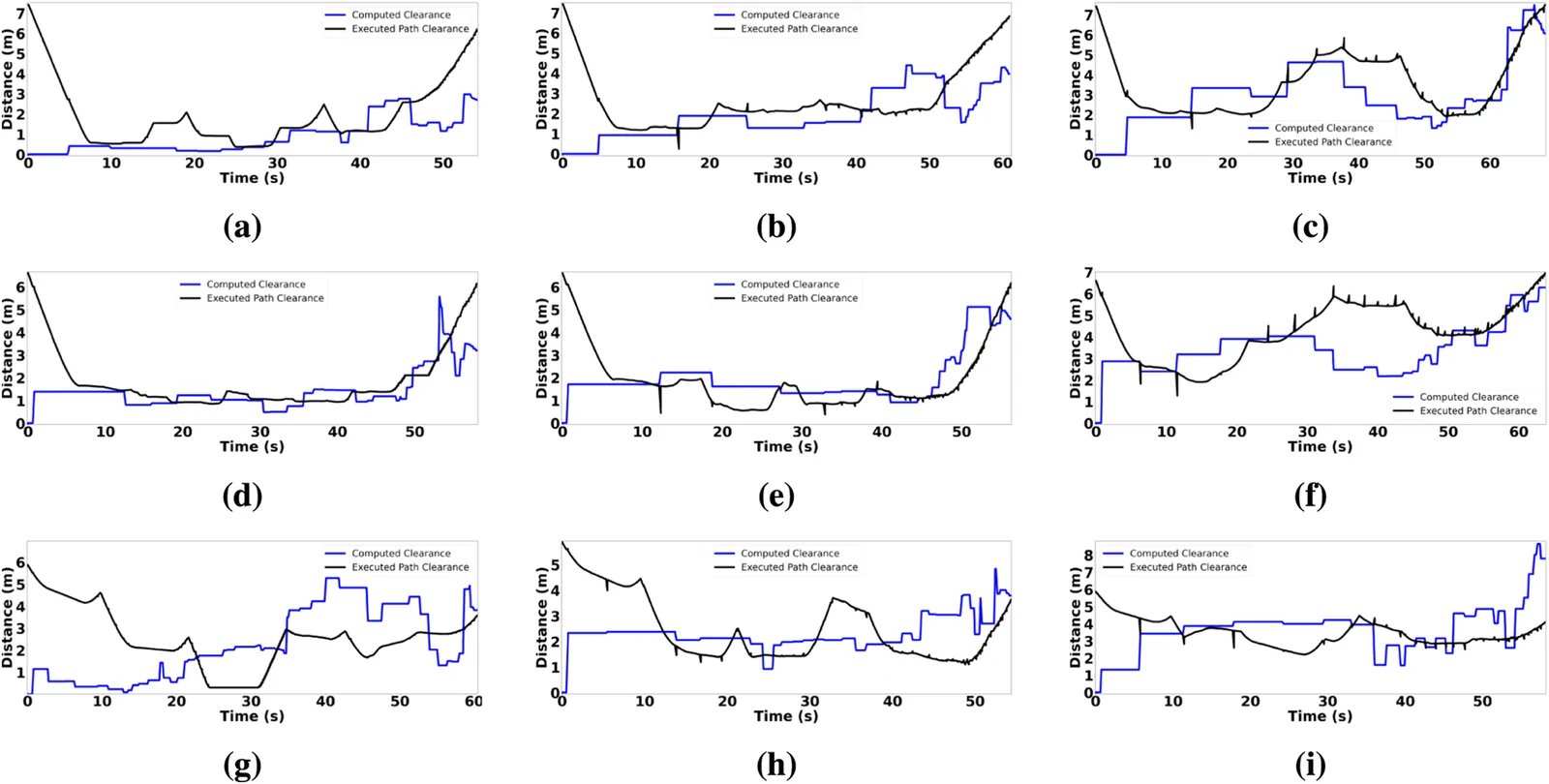
The plots for the computed clearance (blue) with the actual clearance during the execution (black) for environments *m1*: **(a–c)**, *m2*: **(d–f)**, and *m3*: **(g–i)** (top to bottom) for the Snake-M configuration, under the *Error Free*: **(a,d,g)**, *Medium*: **(b,e,h)**, and *High*: **(c,f,i)** conditions (left to right).

Representative executed trajectories are provided in Figure 5, and the computed and actual clearances during the execution are shown in Figure 6. As can be seen in all cases, the AUV adapts to increased errors by increasing the clearance used for planning, and safer paths are picked. Interestingly, in the high-error case of m1 and m2 (Figures 6c,f), the AUV completely avoided risky local minimum solutions by adapting early to paths maximizing the surrounding free space, when errors due to conditions and unmodeled kinodynamic effects were experienced, showcasing strong safety guarantees. On the other hand, in the other cases, efficiency was favored given that safety was guaranteed for the given conditions in the given environment.

These points are also supported by the corresponding clearance plots of Figure 6 that show a clear trend of increasing the computed clearance in worse conditions, except for m3 . This discrepancy emphasizes the property of the proposed planner to provide safety in dynamic environments. More specifically, in the error-free case (Figure 6g), the clearance was initially lower than the other two cases (Figures 6h,i), until changes in the motion of surrounding obstacles increased the clearance, forcing the AUV to quickly maneuver, which increased the effect of unmodeled dynamics. Nevertheless, with worst-case reasoning, the clearance was increased appropriately for the duration of this high-risk area to safely navigate the AUV. It is worth noting that often the clearance of the executed trajectory might be smaller than the computed trajectory. This is a normal key effect of the proposed methodology, given that the increased clearance provides tight bounds that mitigate expected diversions from the planned path, and no collisions are encountered as long as the executed clearance remains positive.

**FIGURE 7:**
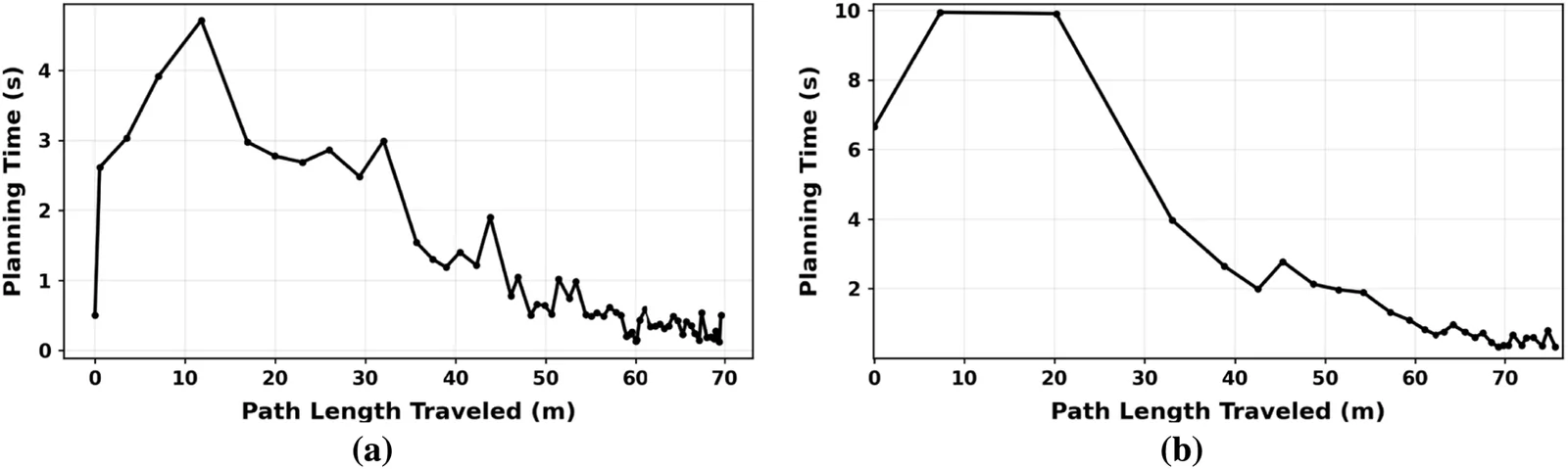
Example instances of the Snake-M in high-error conditions in the m3 map using ResiPlan navigation **(a)** and in the m5 map using ResiVis active perception in **(b)**. Both instances are from the cases with the largest replanning times and largest variances. With the proposed technique, replanning times reach even 10 s at the start due to optimization of the entire path (more than 70 m in length), while consistently in all cases less than 15 m, replanning takes less than 1 s.

The aggregated replanning times for all experiments, with 10 trials each, are shown in Table 1, compared with the non-adaptive baseline. Table 2 provides the percentage of trials with collisions. Despite the computational challenge of the problem, the average replanning time remained approximately 1 s for paths expanding up to 80 m, indicating the real-time or near-real-time capacity of the proposed framework even for complex kinodynamic systems. On average, higher error conditions lead to higher replanning times, with any exceptions being a result of the current pushing the platform in easier free-space areas. To provide further insights into the high deviation of the replanning times, we provide two representative cases from two test cases with the largest mean replanning times and variances in Figure 7. The path length affects the computation time of the solution, indicating a potential shortcoming of the proposed methodology toward some instability in the replanning times and increasing them in conditions that necessitate larger clearance, thus also longer paths. However, it is consistently decreasing quickly. For paths shorter than 15 m, even in the most challenging cases, replanning was taking less, or significantly less, than 1 s. At the same time, please note that the main objective of this work is to showcase the proposed concepts and their potential. Thus, no explicit effort was made toward improving efficiency further in this work, and it is left for future work. Excluding software optimization, simple solutions that would provide fast replanning could be implementing a receding horizon approach as in previous works (Amundsen et al., 2024a; Bjørlo et al., 2025), which is also more appropriate in the harsh underwater conditions due to turbidity, as well as warm-starting of the optimization with state-of-the-art fast sampling-based techniques (Wilson et al., 2025).

**TABLE 1:** Average replanning times from 10 iterations for the proposed autonomous navigation and inspection methods compared to the baseline with a constant clearance in different maps and error conditions.

		Average replanning times (s)					
Method		Proposed method			Baseline (constant $d_{i}^{\mathrm{safe}}=0.5$ m)		
Conditions		Error free	Medium	High	Error free	Medium	High
Snake-M navigation	m1	0.28 ± 0.62	0.69 ± 1.28	0.84 ± 1.45	0.10 ± 0.25	0.49 ± 0.86	0.57 ± 0.89
	m2	0.29 ± 0.59	0.59 ± 1.11	0.68 ± 1.21	0.07 ± 0.06	0.51 ± 0.54	0.55 ± 0.63
	m3	0.47 ± 0.46	0.72 ± 1.06	1.92 ± 2.18	0.35 ± 0.37	0.41 ± 0.39	0.48 ± 0.47
Snake-M inspection	m4	1.22 ± 1.63	1.37 ± 1.78	1.21 ± 1.74	1.38 ± 1.85	1.40 ± 1.74	1.28 ± 1.92
	m5	1.37 ± 1.70	1.69 ± 2.22	1.67 ± 2.17	1.39 ± 1.86	1.33 ± 1.90	1.26 ± 2.06
ROV-UVMS inspection	m6	0.59 ± 0.75	0.90 ± 1.21	1.16 ± 1.30	0.61 ± 0.73	0.51 ± 0.65	0.45 ± 0.61
	m7	0.50 ± 0.70	0.40 ± 0.62	0.45 ± 0.73	0.44 ± 0.73	0.42 ± 0.68	0.32 ± 0.57

The red color indicates that collisions occurred in some trials. Note that, despite the increased replanning times, only a single collision occurred with the proposed technique.

**TABLE 2:** Percentages of runs in collision from 10 iterations for the proposed autonomous navigation and inspection methods compared to the baseline with a constant clearance in different maps and error conditions.

		Percentage of runs with collisions (%)					
Method		Proposed method			Baseline (constant $d_{i}^{\mathrm{safe}}=0.5$ m)		
Conditions		Error free	Medium	High	Error free	Medium	High
Snake-M navigation	m1	**0**	**0**	**0**	**0**	30	100
	m2	**0**	10	**0**	**0**	100	100
	m3	**0**	**0**	**0**	100	**0**	80
Snake-M inspection	m4	**0**	**0**	**0**	**0**	100	100
	m5	**0**	**0**	**0**	**0**	100	100
ROV-UVMS inspection	m6	**0**	**0**	**0**	**0**	**0**	40
	m7	**0**	**0**	**0**	10	70	100

No collisions are indicated with bold 0 for clarity, while 100 indicates collisions in every run. The proposed adaptive methods consistently produced safer performance under all trials, in contrast to the baseline.

Regarding safety, the proposed adaptive methodology provides consistently safe solutions in contrast to the baseline. Of all the trials, we noted a minor collision that occurred in one trial when a large “unexpected” error occurred after a past experience of abnormally low errors, which also indicated an interesting and important attribute of the proposed framework. To clarify further, ResiPlan and ResiVis are adaptively holistic and agnostic pipelines and rely on the discrepancy between the expected and the measured placement of the robot as a way to infer the system and environment uncertainty and adapt in the observed conditions. In that specific case, the pipeline adapted to perceived fewer erroneous conditions and could not react in time to the sudden displacement, with the added replanning delay of finding a much more conservative path.

### 5.3 Active perception simulations

We validate active perception in both simulated platforms depicted in Figure 3 with different sensing configurations in challenging environments. For the simulated Snake-M AUV, two sensors are placed on the sides around the centroid of the middle link, with a desired sensing range of 2 m, to study its capacity to produce paths that are safe while the platform senses the objectives from close proximity. This configuration was tested in two environments:

- *m4* represents a static infrastructure inspection task, such as an underwater pipe, with two support columns.
- *m5* simulates a deformable infrastructure inspection task, similar to aquaculture inspection missions or disaster scenarios. To increase the challenge, 21 poles were used to represent the target surface with size 1×1×15 m^3^ each, which are synchronously moving in a horizontal repetitive pattern, with a constant velocity of 0.1 m/s at a range of 4 m, simulating a wavy pattern in the target structure.

For the simulated M-ROV, sensors of the same characteristics were placed on the front of the main frame and at the end effector of the manipulator. To validate multi-objective active perception, the forward-looking sensor was assigned to face the next way point, and the end-effector sensor to focus on the objects of interest. This setup was tested in two environments:

- *m6* consists of randomly arranged pillars, such as in a disaster scenario, with a pipe-like structure as an inspection objective, and two other disconnected objectives: a static box at the start of the experiment and a dynamic one executing a bounded random path at a velocity of 0.1 m/s near the goal.
- *m7* represents an aquaculture inspection scenario, in which the M-ROV is following along a wall moving horizontally in a repetitive pattern at a range of 4 m with 0.1 m/s. The sensing targets are placed on the wall at different depths, forcing the M-ROV to use both the manipulator and the main base to observe the targets. Additionally, four objects of the same size as the M-ROV move towards the robot at 0.1 m/s in a formation that requires a change of depth for avoidance. Such a situation could take place if the AUV coexists with other sensors or robots in the facility.

**FIGURE 8:**
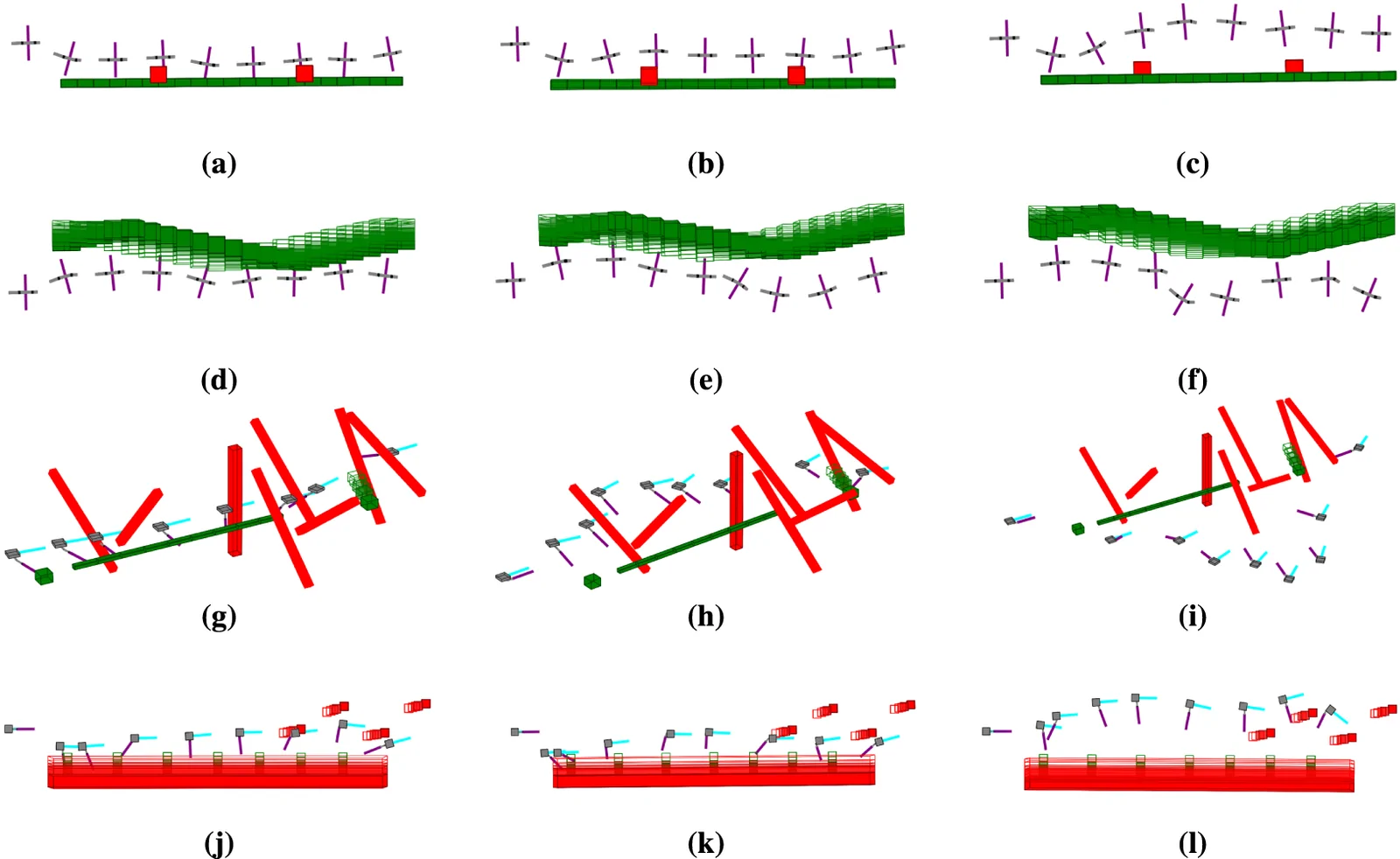
The snapshots of executions in the test environments for ResiVis applied on Snake-M **(a–f)** and the ROV-UVMS **(g–l)**, in the environments *m4*–*m7*, under *Medium* conditions. Obstacles are shown in red, and objects of interest are shown in green, with the fading pattern indicating their motion during the first 20 s. The purple lines on the sides of Snake-M indicate the sensing line segments modeled, while for ROV-UVMS, the purple line indicates the sensing line segment for the end-effector sensor. The cyan line indicates the front sensor on the main body.

**FIGURE 9:**
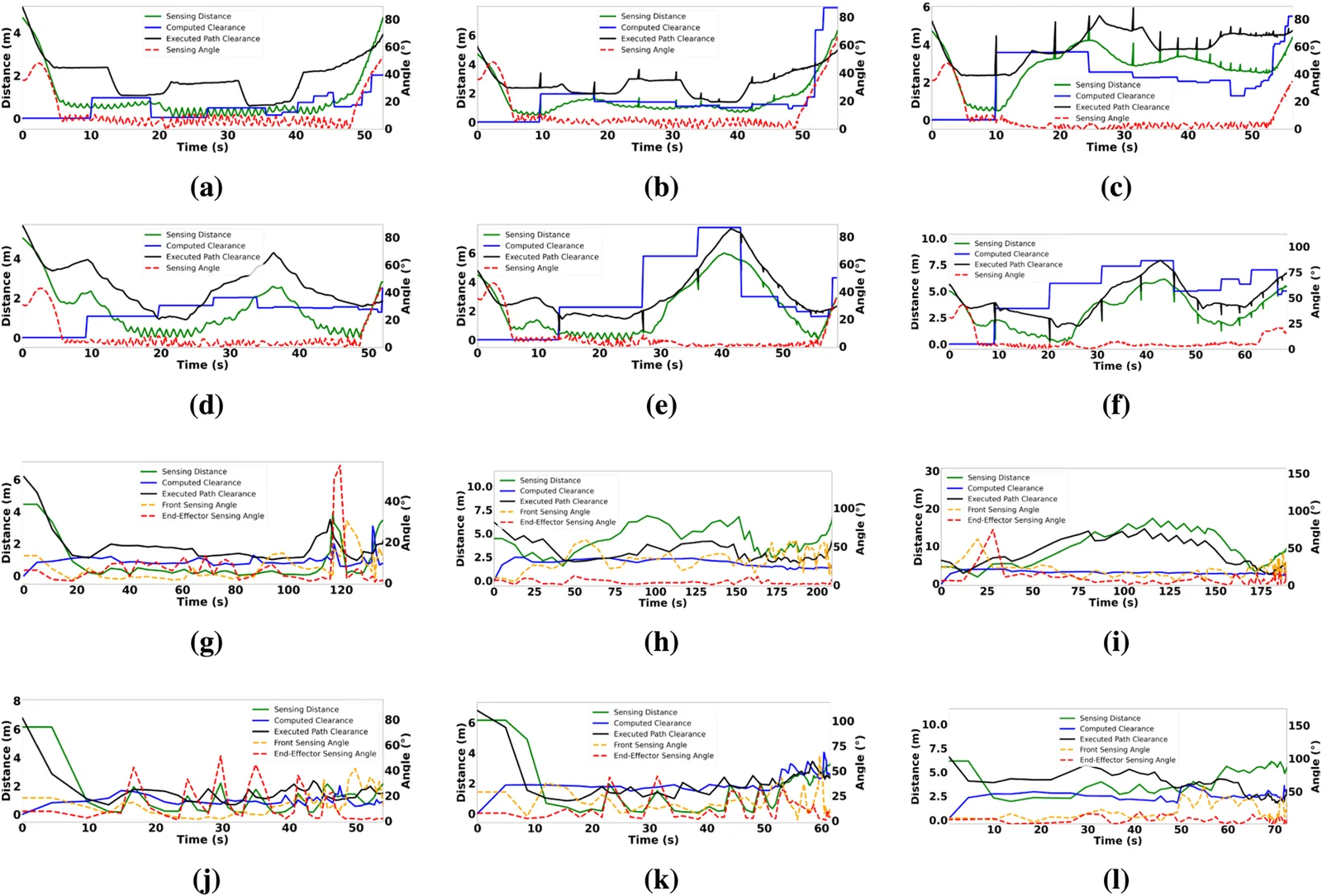
The plots for visibility, shown in the form of sensing distance (green) and sensing angle error (red and orange), during execution for environments *m4*–*m7*, under *Error Free*, *Medium*, and *High* conditions (left to right), for both the Snake-M **(a–f)** and ROV-UVMS **(g–l)** cases. The sensing angle error (orange and red) corresponds to the right-hand axis in each plot. The sensing error angles are shown on the right-hand axis in each plot, and the computed and actual clearances are shown in blue and black, respectively.

The representative executed trajectories are provided in Figure 8. Figure 9 shows the corresponding plots with the computed clearance, the actual clearance, the sensing distance of desired objectives, and the sensing angle from the center of the sensor. The units for the angles are shown on the right side.

Similar to the experiments of the previous paragraph, higher errors lead to more conservative performance in all cases.

In most cases, the AUVs were able to perceive the target structures within the desired distance, with spikes in angular errors noted only momentarily, due to switching between different sensing objectives. However, in high-error cases, or when obstacles are lying in between, the solutions drove the underwater vehicles further from the structures, showcasing that safety is prioritized over observations. This is also illustrated in Table 2, with no collisions occurring in any of our tests, while the baseline with the constant clearance produced more risky paths that led to a high percentage of collisions.

With the addition of the sensing constraints, there is an increase (up to five times) in the corresponding replanning times reported in Table 1, indicating a bottleneck for robust real-time performance using the current implementation. We provide potential ways to address this issue in the previous paragraph.

The increased replanning times not only affected the real-time capacity of the proposed planner but also increased the desired clearances, given that, within a single replanning cycle, the robots were exposed to more challenging conditions, increasing the measured errors due to drifting. At the same time, the proposed framework shows its strong capacity to simultaneously adapt to the challenging conditions, unknown kinodynamics, and the potential challenges introduced by the method itself. This further emphasizes its holistic nature, enabling robust agnostic and adaptive decision making by both monitoring and mitigating external factors and by self-monitoring and self-mitigation of its own shortcomings.

**FIGURE 10:**
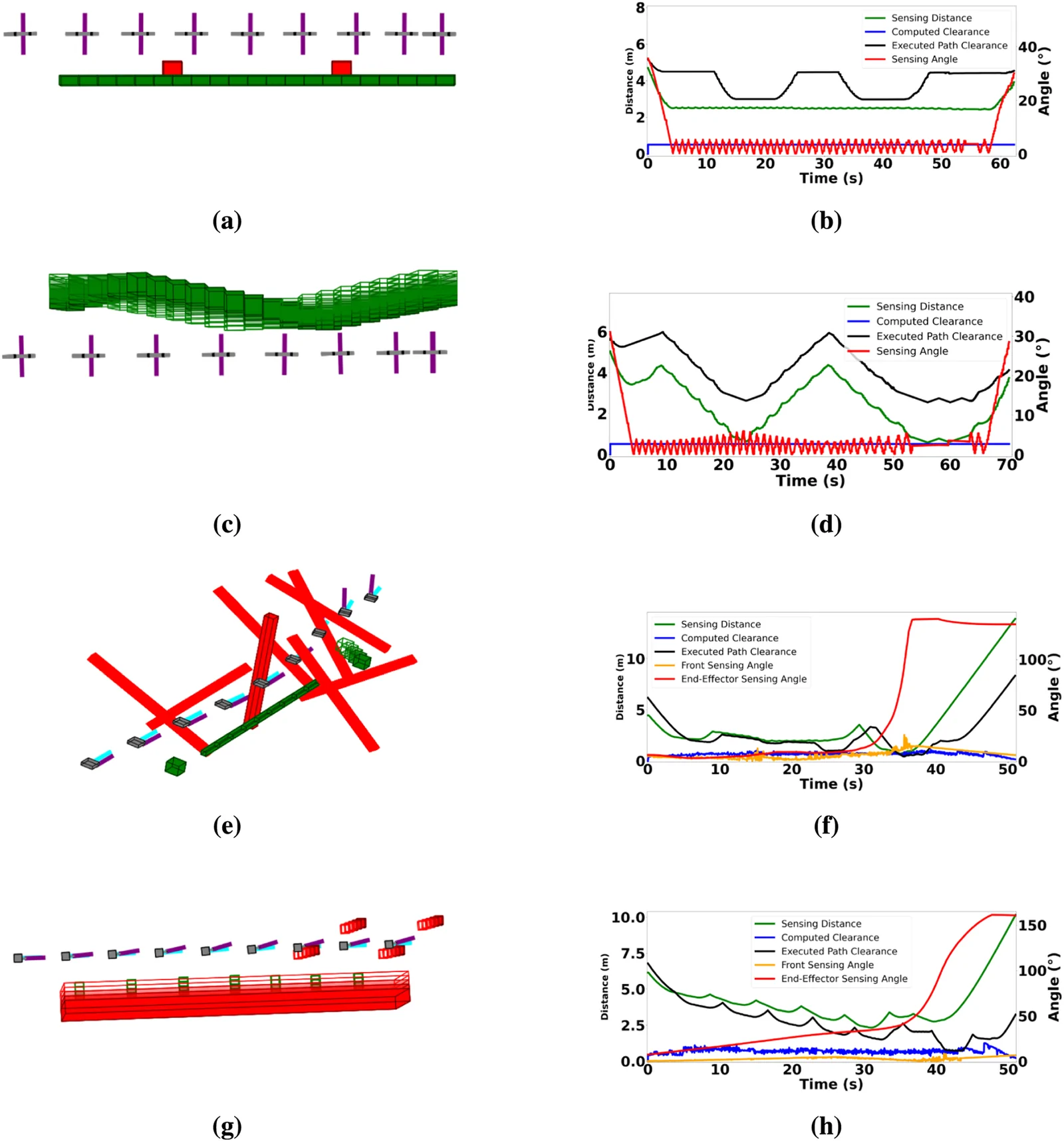
Path instances of ResiPlan, the proposed navigation pipeline, on the environments used for active perception (m4: **(a,b)**, m5: **(c,d)**, m6: **(e,f)**, and m7: **(g,h)**) from top to bottom, respectively, along with the corresponding plots for *Error Free* conditions. Without the proposed active perception constraints, the AUVs ignore the desired objects to be inspected, which leads to perceiving them both from further away and from large angles, in the case of the ROV-UVMS.

Finally, as shown in Figure 10, to validate the contribution of the active perception modules, we performed additional experiments corresponding to the environments *m4*–*m7* and error-free conditions of panels a, d, g, and j of Figures 8, 9 by disabling the active perception constraints. For the Snake-M configuration, no significant differences were observed in the angular sensing error, given that the target structure already lies on the side of the robot, although there is a significant increase in the sensing distance with respect to ResiVis. In the case of the ROV-UVMS, despite the significant increase in the sensing distance, as expected, the sensing angle also increased, with the end-effector sensor completely ignoring and, at times, even avoiding sensing the target structures.

## 6 Conclusion

This article contributes, to the best of our knowledge, the first general, adaptive, holistic, and agnostic motion planning framework for enabling safe autonomous navigation and active perception on arbitrary complex articulated underwater robots with arbitrary sensing capacities and unknown kinodynamics in the presence of state uncertainty, motion errors, wave zone conditions, currents, and dynamic obstacles. The new enhancements introduced were validated with challenging simulated experiments that showcased the strong capacity of the proposed methodology to handle autonomous navigation and inspection queries with significant challenges successfully and safely, while areas of further improvement were identified, specifically improving computational efficiency.

At a higher level, this article serves as a testament to how holistic approaches that embrace generality and remain agnostic to the specifications of the systems considered can scale significantly well and be utilized to solve much more challenging problems.

Future focus will be given toward further validating the contributions on real articulated underwater systems with real deployments, introducing the ability to avoid arbitrary fast-moving dynamic obstacles (Amundsen et al., 2024a), rethinking the problem formulation by introducing learned swept-out transition volumes (Bjørlo et al., 2025), reformulating the active perception constraints to include occlusion-free guarantees, and revisiting the core software of our implementation to increase the replanning frequency (Amundsen et al., 2024b). Finally, we aspire to approach new, exciting, and challenging problems with a holistic mentality, increasing robustness and efficiency beyond the current state of the art.

## Data Availability

The raw data supporting the conclusions of this article will be made available by the authors upon request.

## Footnotes

1
https://www.eelume.com/eelume-m-series
